# Comparison of iodine quantification accuracy on prototype deep silicon photon-counting and energy-integrating detector CT

**DOI:** 10.1007/s00261-025-05098-1

**Published:** 2025-07-28

**Authors:** Aria Salyapongse, Teva Shapiro, Zhye Yin, Scott Slavic, Giuseppe Toia, Meghan Lubner, Timothy Szczykutowicz

**Affiliations:** 1 University of Wisconsin–Madison, Madison, USA; 2 GE HealthCare, Waukesha, USA

**Keywords:** Iodine quantification, Photon-counting, Energy-integrating, Computed tomography, Spectral CT

## Abstract

**Purpose:**

This work examined the iodine quantification accuracy on a prototype deep silicon (dSi) photon-counting detector (PCD) computed tomography (CT) system compared to a rapid kV switching energy-integrating detector (EID) dual-energy (DE) CT system.

**Methods:**

Iodine-containing rods (0–20 mg I/mL) in a phantom (Gammex MECT) were scanned with the prototype dSi PCD and DECT systems. Iodine (water) material density images were made with prototype and commercially available material decomposition algorithms, respectively. Circular regions of interest were placed over the center of the iodine rods to measure iodine accuracy in each slice, and slices were averaged. A correction based on the known issue of background material difference from true water was determined from the 0 mg I/mL (solid water) rod and the relative rod densities and was applied to the iodine quantification. Iodine percent error was defined as the difference between corrected iodine quantification and known iodine quantification, divided by the known iodine, multiplied by 100.

**Results:**

The corrected iodine quantification was within 0.15 mg I/mL for 0–20 mg I/mL iodine rods on the prototype PCD CT and 0.2 mg I/mL for the EID DECT. This translates to iodine percent errors of 0.4–4.3% on the PCD CT and 1.1–76% on the EID DECT for 0.2–20 mg I/mL rods.

**Conclusion:**

Iodine quantification on the prototype dSi PCD CT was within 4.3% for all tested iodine-containing rods, which is similar or better than the performance of the EID DECT system and previous work on the prototype dSi system.

## Introduction

Iodinated contrast media is commonly used in computed tomography (CT) scanning to increase contrast and analyze perfusion or blood flow in single-energy (SE) CT. The quantification of iodine is useful for the characterization of lesions in dual-energy (DE) CT, particularly in abdominal radiology [1–6]. Several studies show that the implementation of DECT into clinical practice improves radiologist diagnostic confidence and accuracy, specifically in the characterization of incidental abdominal lesions and the differentiation between malignant abdominal lymphomas and lymph node metastases [1, 5, 6]. By using iodine density maps and iodine overlap imaging, it is possible to measure iodine content in a voxel of interest [7–9].

Iodine quantification requires spectral data so that a material decomposition algorithm can be applied [10, 11]. This involves measurements at two energies (for DE CT) that are typically transformed into an iodine and water basis system to quantify the iodine component of a given tissue or region of interest (ROI) [10, 11]. Photon-counting detector (PCD) CT works similarly, except that only a single measurement is needed because the detector is energy-resolving [12], but the energy information is still transformed into the iodine and water basis system to quantify iodine.

The current methods of iodine quantification in multi-energy CT rely on two different detector technologies: energy-integrating detectors (EID) and photon-counting detectors (PCD). Although there are several different implementations of EID DECT (e.g., dual-source, fast-kV switching, rotate-rotate, TwinBeam, and dual-layer systems), the energy resolving nature of the EID DECTs is the same [11]. EID CTs convert x-rays into light and then the light into an electrical signal via scintillators and light-sensitive electronics, respectively, and the PCDs convert x-rays directly into electrical signal via a semiconductor [12].

While there are several studies demonstrating the iodine quantification accuracy on the commercially available PCD CT system (NAEOTOM Alpha, Siemens Medical Solutions, Malvern PA) [13–16] and a preliminary iodine sensitivity study on this prototype [17], there is not yet similar quantification for the prototype deep silicon (dSi) PCD CT system (GE HealthCare, Waukesha, WI) with a full comparison to current literature. In the past, this system has been shown to provide more accurate and consistent measurements of CT numbers for both patient size and mispositioning [17–22] when compared to EID CT. Therefore, this dSi PCD CT system may also provide increased iodine accuracy. The purpose of this work is to investigate the iodine quantification accuracy of a prototype dSi PCD CT [17–22].

## Methods

### Investigators

ZY and SS are employees of GE HealthCare. The vendor authors assisted in data collection and performed reconstruction for the dSi PCD CT data. The remaining authors, who are not employees of the vendor, had full control of the processing of all data and all information submitted for publication.

### Phantom

The phantom used in this work was a Gammex multi-energy CT (MECT) phantom (MECT Phantom, Sun Nuclear Corporation, Middleton, WI) [23] which has a solid water background and several rod inserts of different materials. The rod inserts used in this work were the solid water rod and the iodine-containing rods of 0.2, 0.5, 1, 2, 5, 10, 15, and 20 mg I/mL with diameters of 2.8 cm.

### Imaging protocol

The MECT phantom was scanned on both a commercially available DE EID CT (Discovery CT750 HD, GE HealthCare, Waukesha, WI) and a prototype dSi PCD CT system from GE HealthCare (Waukesha, WI). The DE EID CT utilized fast 80/140 kV switching (Gemstone Spectral Imaging) mode, 600 mA, 5 mm slices, 0.8 s rotation time, 0.981:1 pitch, and 40 mm collimation helical scanning, resulting in a CTDI_vol_ of 25.53 mGy. The PCD CT scan utilized 120 kV, 600 mA, 0.8 s rotation time, 0.42 mm slice thickness, and 40 mm collimation axial scanning, resulting in a CTDI_vol_ of 32.85 mGy. The phantom was positioned with its geometric center at isocenter for all scans.

### Image reconstruction

The prototype dSi PCD CT uses eight energy bins, with three dynamic bins below a 33 keV energy level to collect Compton scattered photons (i.e., low energy photons generated inside the detector from Compton interactions which if left un-recorded would decrease dose efficiency and energy resolution), and five static bins at 33, 44, 52, 60, and 80 keV [24]. The PCD CT iodine (water) images were reconstructed at GE HealthCare in a 20 cm field-of-view (FOV). Pulse pileup corrections were applied to the raw data, which was then put into a prototype material decomposition algorithm specific to the prototype scanner to obtain the iodine map images.

The DE EID CT iodine (water) images were reconstructed in a 40 cm FOV with commercially available, FDA-cleared material decomposition software (Discovery CT750 HD, GE HealthCare, Waukesha, WI) to generate iodine maps.

### Image analysis

Circular ROIs of 120 mm^2^ were placed on each slice in the iodine (water) image series to generate a mean iodine quantification for each rod. Slices were averaged together to generate a final average iodine quantification value and standard error.

Based on previous work, it is known that there is an inherent iodine density error on the quantification of iodine in the Gammex MECT phantom due to the difference between solid water and true water [10]. Therefore, a correction based on this previous work was applied to the raw iodine quantification on iodine (water) images. This correction is based on the knowledge that due to the chemical composition of the solid water, the solid water background will have a non-zero iodine density in an iodine density image. The iodine error on the solid water (0 mg I/mL iodine density) rod was scaled by the volume fraction ($\:{VF}_{SW}$) (Eq. 1) of solid water present in each of the iodine rods and then subtracted from the raw iodine density values (Eq. 2).

1 $$\:{VF}_{SW}=\frac{{\rho\:}_{I}-{\rho\:}_{rod}}{{\rho\:}_{I}-{\rho\:}_{SW}}$$

In Eq. 1, $\:{\rho\:}_{rod}$ is the mass density of the iodine rod, $\:{\rho\:}_{SW}$ is the mass density of the solid water rod, $\:{\rho\:}_{I}$ is the mass density of elemental iodine, and $\:{VF}_{SW}$ is the volume fraction of solid water in a rod. $\:{\rho\:}_{rod}$ and $\:{\rho\:}_{SW}$ are provided by the phantom vendor, and $\:{\rho\:}_{I}$ is taken from physical properties table of reference 26 [25].

2 $$\:{\rho\:}_{I,corr}={\rho\:}_{I,raw}-{VF}_{SW}\cdot\:{\rho\:}_{I,SW}$$

In Eq. 2, $\:{\rho\:}_{I,corr}$ is the corrected density of the iodine measurement, $\:{\rho\:}_{I,raw}$ is the raw density iodine density measurement from the iodine (water) images, and $\:{\rho\:}_{I,SW}$ is the iodine density measurement from an iodine (water) image of the solid water rod.

This correction is intended to account for the error introduced due to the solid water background being different from the true water that is assumed in an iodine (water) material decomposition.

Once corrected iodine density values were determined, the iodine density percent error was calculated according to Eq. 3 by comparing to the phantom vendor nominal iodine mass densities ($\:{\rho\:}_{I,nominal}$):

3 $$\:Percent\:error=\:\frac{{\rho\:}_{I,nominal}-{\rho\:}_{I,corr}}{{\rho\:}_{I,nominal}}$$

T-tests were performed on the corrected iodine density value errors both PCD and EID CT quantifications to evaluate if the errors from the two systems were significantly different from zero.

### Literature comparison

To contextualize the results within current iodine quantification literature, it was important to compare the iodine quantification to a wide range of results from other scanners and phantoms. When the phantom used in the comparative literature was iodine or sodium iodide dissolved in water, a comparison was made to the corrected iodine density results because of the known issue with the solid water background. When the phantom used in the comparative literature was the same phantom (Gammex MECT, Sun Nuclear), a comparison was made to the raw iodine density results because the same solid water background issue would be present in those studies. Comparisons were made between the iodine density quantification accuracy results in this work and both commercially available EID and PCD CT systems.

## Results

The raw iodine density values (i.e., the iodine densities determined from the iodine density maps without the solid water density correction) are show in Table 1, column 4 and the corrected iodine density values are in column 6.

**Table 1:** Raw and corrected iodine density values

Nominal Iodine Density [$\:\frac{\boldsymbol{m}\boldsymbol{g}\:\boldsymbol{I}}{\boldsymbol{m}\boldsymbol{L}}]$	Rod Density [$\:\frac{\boldsymbol{m}\boldsymbol{g}}{\boldsymbol{m}\boldsymbol{L}}$]	Volume Fraction $\:{VF}_{SW}=\frac{{\rho\:}_{I}-{\rho\:}_{rod}}{{\rho\:}_{I}-{\rho\:}_{SW}}$	Raw Iodine Density [$\:\frac{\boldsymbol{m}\boldsymbol{g}\:\boldsymbol{I}}{\boldsymbol{m}\boldsymbol{L}}]$		Error calculation [$\:\frac{\boldsymbol{m}\boldsymbol{g}\:\boldsymbol{I}}{\boldsymbol{m}\boldsymbol{L}}]$ $\:{VF}_{SW}\bullet\:{\rho\:}_{I,SW}$		Corrected Iodine Density [$\:\frac{\boldsymbol{m}\boldsymbol{g}\:\boldsymbol{I}}{\boldsymbol{m}\boldsymbol{L}}]$	
			EID	PCD	EID	PCD	EID	PCD
0 (solid water)	1.021	1	0.20	0.11	$\:1\bullet\:0.20$	$\:1\bullet\:0.11$	0	0
0.2	1.026	0.9987	0.25	0.30	$\:0.9987\bullet\:0.20$	$\:0.9987\bullet\:0.11$	0.0465	0.194
0.5	1.026	0.9987	0.53	0.63	$\:0.9987\bullet\:0.20$	$\:0.9987\bullet\:0.11$	0.33	0.5175
1	1.026	0.9987	1.04	1.15	$\:0.9987\bullet\:0.20$	$\:0.9987\bullet\:0.11$	0.836	1.043
2	1.027	0.9985	2.04	2.13	$\:0.9985\bullet\:0.20$	$\:0.9985\bullet\:0.11$	1.84	2.02
5	1.030	0.9977	5.03	5.10	$\:0.9977\bullet\:0.20$	$\:0.9977\bullet\:0.11$	4.82	4.98
10	1.034	0.9967	10.00	10.00	$\:0.9967\bullet\:0.20$	$\:0.9967\bullet\:0.11$	9.79	9.90
15	1.039	0.9954	14.93	14.95	$\:0.9954\bullet\:0.20$	$\:0.9954\bullet\:0.11$	14.73	14.84
20	1.043	0.9944	19.98	19.97	$\:0.9944\bullet\:0.20$	$\:0.9944\bullet\:0.11$	19.78	19.87

EID = energy-integrating detector, PCD = photon-counting detector

To clarify how the correction works, an example calculation is illustrated below. The first step is to determine the volume fraction of solid water in each insert in the phantom, which can be accomplished using the user manual of the Gammex MECT phantom [17] to obtain the physical properties of the insert and Eq. 1:

$$\:{VF}_{SW}=\frac{{\rho\:}_{rod}-{\rho\:}_{SW}}{{\rho\:}_{I}-{\rho\:}_{SW}}=\frac{1.021\raisebox{1ex}{$mg$}\!\left/\:\!\raisebox{-1ex}{$mL$}\right.-1.026\:\raisebox{1ex}{$mg$}\!\left/\:\!\raisebox{-1ex}{$mL$}\right.}{4.93\:\raisebox{1ex}{$mg$}\!\left/\:\!\raisebox{-1ex}{$mL$}\right.-1.021\:\raisebox{1ex}{$mg$}\!\left/\:\!\raisebox{-1ex}{$mL$}\right.}=0.9987$$

Once the volume fraction of solid water is known, it can be multiplied by the density of iodine found in the solid water insert (0.20 $\:\frac{mg\:I}{mL}$ for EID and 0.11 $\:\frac{mg\:I}{mL}$ for PCD) to obtain the error on iodine density:

$$\:{VF}_{SW}\bullet\:{\rho\:}_{I,SW}=0.9987\bullet\:0.20=0.1997$$

Once the error is known, the corrected iodine density can be obtained using Eq. 2:

$$\:{\rho\:}_{I,corr}={\rho\:}_{I,raw}-{VF}_{SW}\bullet\:{\rho\:}_{I,SW}=0.25-0.1997=0.05$$

**Fig. 1:**
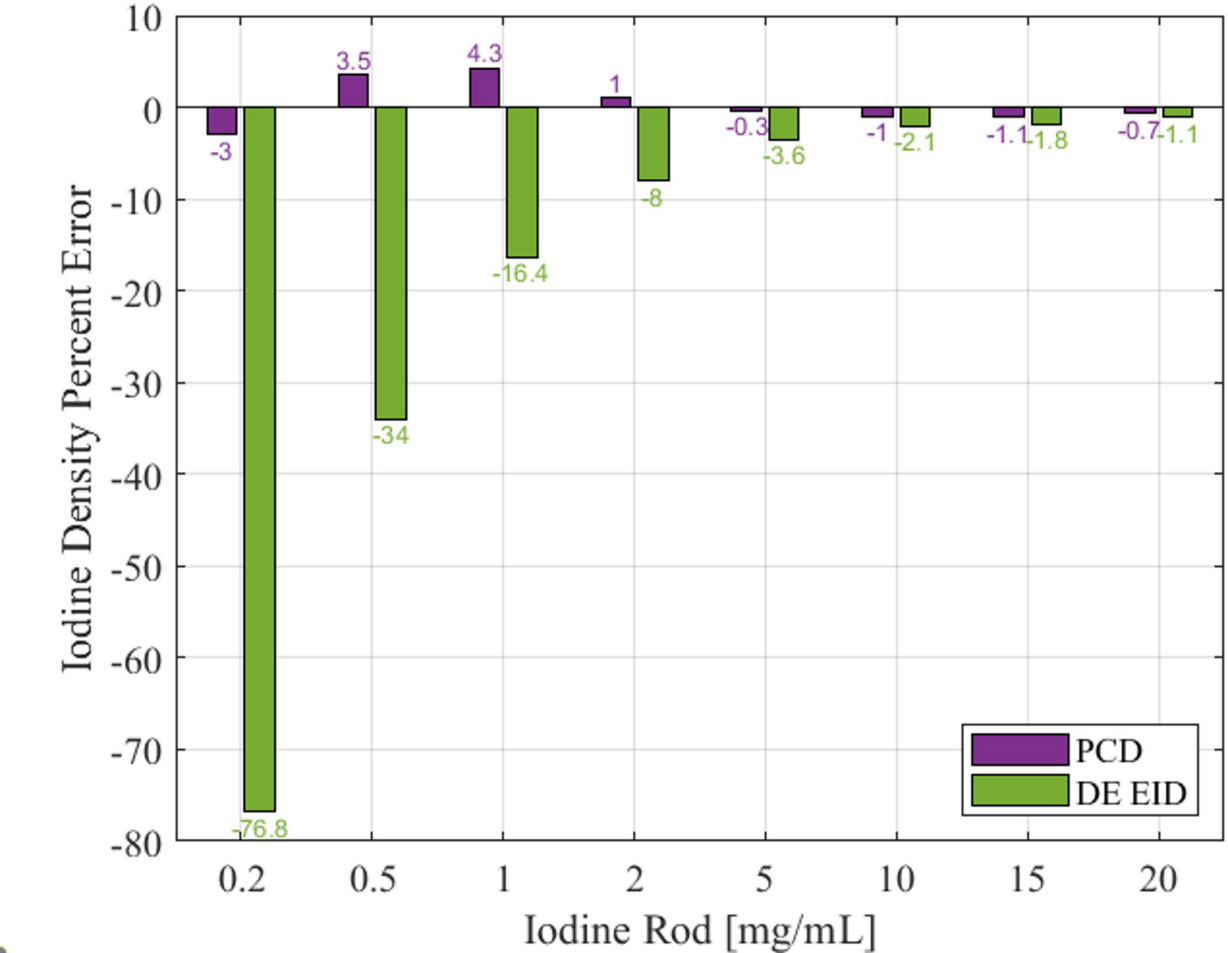
demonstrates the iodine density percent error for both DE EID CT and PCD CT results

Figure 1 Percent density error of the iodine quantification with the deep silicon photon-counting detector (PCD) CT and the dual-energy (DE) energy-integrating detector (EID) CT.

With the correction, the iodine density percent error is less than 5% for all iodine values tested for the dSi PCD CT system. Iodine density percent error is less than 20% for iodine values between 1 and 20 mg I/mL for the EID CT system but up to 77% for iodine values less than 1 mg I/mL. T-tests on the corrected iodine densities demonstrate that there is not a significant difference between the PCD CT iodine quantification errors and zero (*p* = 17), but there is a significant difference between the EID CT iodine quantification errors and zero (*p* < 0.001).

**Fig. 2:**
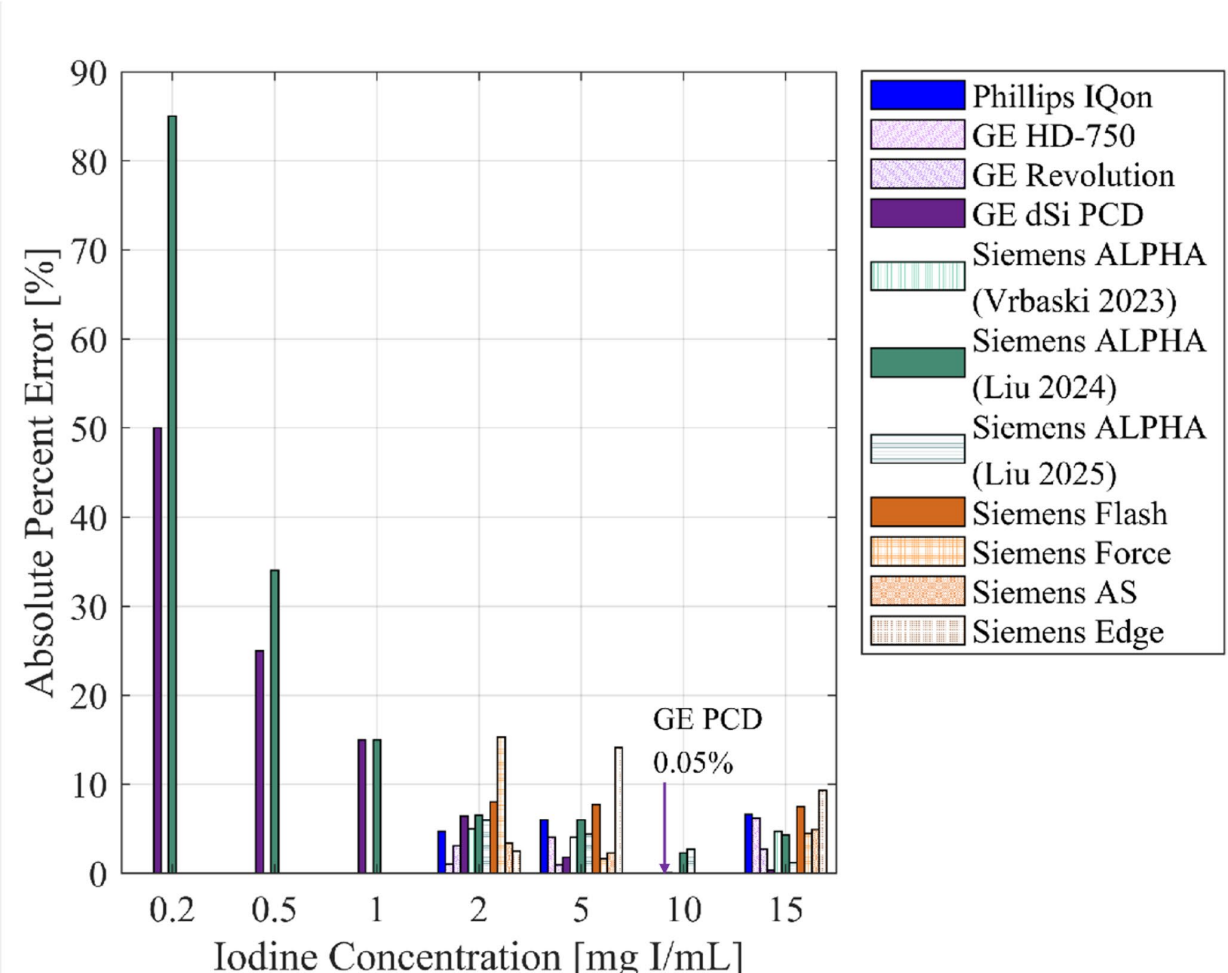
Graphical representation of the literature comparison results in Table 2

Table 2; Fig. 2 demonstrate a comparison between the iodine quantification on current work and iodine quantification of previous works.

**Table 2:** Literature comparison between present study PCD results and literature on iodine quantification with spectral CT

Publication	Scanner	Phantom	Iodine concentrations [$\:\frac{\boldsymbol{m}\boldsymbol{g}\:\boldsymbol{I}}{\boldsymbol{m}\boldsymbol{L}}$]	% Error	Present study PCD % error
Jacobsen et al., 2017 [11]	GE HD-750 80/140 kV	MECT Gammex	2	0.98^a^	6.5^b^
	GE Revolution CT 80/140 kV			3.1^a^	
	Phillips IQon 120 kV			4.7^a^	
	Siemens Flash 80/140Sn kV			−8.0^a^	
	Siemens Force 90/150 Sn kV			15.3^a^	
	Siemens AS 128 80/140 kV			3.4^a^	
	Siemens Edge 120AuSn kV			−25^a^	
	GE HD-750 80/140 kV		5	−4.0^a^	2.0^b^
	GE Revolution CT 80/140 kV			−0.93^a^	
	Phillips IQon 120 kV			6.0^a^	
	Siemens Flash 80/140Sn kV			−7.7^a^	
	Siemens Force 90/150 Sn kV			1.6^a^	
	Siemens AS 128 80/140 kV			−2.3^a^	
	Siemens Edge 120AuSn kV			−14.1^a^	
	GE HD-750 80/140 kV		15	−6.2^a^	−0.33^b^
	GE Revolution CT 80/140 kV			−2.7^a^	
	Phillips IQon 120 kV			6.6^a^	
	Siemens Flash 80/140Sn kV			−7.5^a^	
	Siemens Force 90/150 Sn kV			−4.5^a^	
	Siemens AS 128 80/140 kV			−4.9^a^	
	Siemens Edge 120AuSn kV			−9.3^a^	
Vrbaski et al., 2023 [13]	Siemens NAEOTOM Alpha 140 kV Standard Dose	MECT Gammex Medium	2	5^a^	6.5^b^
			5	4^a^	2.0^b^
			15	4.7^a^	−0.33^b^
Ren et al., 2024 [14]	Siemens NAEOTOM Alpha 120 kV	Iohexol + Water (30 cm)	5	0	−0.32^c^
			10	3.0	−1.0^c^
			15	2.0	−1.1^c^
Liu et al., 2024 [15]	Siemens NAEOTOM Alpha 120 kV Medium Dose	MECT Gammex	0.2	−85	50^b^
			0.5	−34	25^b^
			1	−15	15^b^
			2	−6.5	6.4^b^
			5	−6.0	1.8^b^
			10	−2.3	0.05^b^
			15	−4.3	−0.34^b^
Liu et al., 2025 [16]	Siemens NAEOTOM Alpha 120 kV Large Dose	MECT Gammex	2	−6	6.4^b^
			5	4.4	1.8^b^
			10	2.7	0.05^b^
			15	1.2	−0.34^b^
Crotty et al., 2022 [17]	GE Prototype dSi PCD CT	Iohexol + Water	0.25	4	−3.0^d^

^a^Indicates that the exact bias or iodine quantification were not provided and thus were estimated based on the provided figures. ^b^Indicates raw iodine density percent error values because the comparative literature also used a solid water-based phantom. ^c^Indicates corrected raw iodine density percent error values because the comparative literature used dissolved iodine or sodium iodine. ^d^Indicates that the same iodine concentration was not available and therefore the closest iodine concentration was used as comparison

## Discussion

This work demonstrates that the iodine density quantification with a physics-motivated correction is more accurate or similarly accurate on the dSi photon-counting detector (PCD) CT compared to the commercially available energy-integrating detector (EID) CT. The dSi PCD CT exhibited an iodine density percent error less than 5% for all iodine densities tested, while the EID CT exhibited an iodine density percent error from 1 to 77% across the tested iodine densities.

Jacobsen et al. (2018) published work that examined iodine quantification on several EID systems, which demonstrated a percent iodine error of −25–15.3% for 2 mg I/mL, −14.1–6.0% for 5 mg I/mL, and − 9.3–6.6% for 15 mg I/mL [11]. In the present work these iodine concentrations (2, 5, and 15 mg I/mL) yielded a percent error of 6.5%, 2.0%, and − 0.33%. In this comparison, between the commercially available EID systems and the current prototype dSi PCD CT, the PCD CT performs similarly for 2 mg I/mL, and better for 5 and 15 mg I/mL. Vibraski et al. (2023) measured iodine quantification accuracy using the MECT Gammex phantom and a commercially available PCD CT scanner (Siemens NAEOTOM Alpha, Siemens Medical Solutions, Malvern, PA), and demonstrated iodine density percent errors of 5%, 4%, and 4.7% for 2, 5, and 15 mg I/mL, respectively [13]. In comparison, this work demonstrated iodine density percent errors of 6.5%, 2.0%, and − 0.33% respectively, which reveals slightly higher errors at low iodine concentrations (2 mg I/mL) and better performance for larger iodine concentrations (5 and 15 mg I/mL). Similarly, Liu et al. (2024) also used the Siemens NAEOTOM Alpha scanner and the MECT Gammex phantom to investigate iodine quantification, resulting in iodine density percent errors of −85%, −34%, −15%, −6.5%, −6.0%, −2.3%, and − 4.3% for iodine concentrations of 0.2, 0.5, 1, 2, 5, 10, and 15 mg I/mL, respectively [15]. In the current work with the same iodine concentrations, the iodine density percent errors were found to be 50%, 25%, 15%, 6.4%, 1.8%, 0.05%, and − 0.34%, which are either on par with the results from Liu et al. (2024) or better [15]. Liu et al. (2025) also utilized the NAEOTOM Alpha scanner and Gammex phantom to investigate iodine quantification error, with iodine density percent errors of −6%, 4.4%, 2.7%, and 1.2% for iodine concentrations of 2, 5, 10, and 15 mg I/mL, respectively [16]. For the same iodine concentrations in this work, the percent density errors were found to be 6.4%, 1.8%, 0.05%, and − 0.34% [16]. These results are slightly worse than Liu et al. (2025) for the 2 mg I/mL iodine concentration, but better for the 5, 10, and 15 mg I/mL concentrations. Finally, Ren et al. (2024) made a phantom using iodinated-contrast media (Omnipaque 350, GE HealthCare, Princeton, NJ) and purified water, with iodine concentrations of 5, 10, and 15 mg I/mL and iodine density percent errors of 0%, 3.0%, and 2.0% [14]. In comparison, the corrected iodine density errors (corrected because with a water-iodine phantom there is not bias due to the background being solid water [10]) for the same iodine concentrations were − 0.32%, −1.0%, and − 1.1%, which are slightly worse for the smaller iodine concentration (5 mg I/mL) but better for the larger iodine concentrations (10 and 15 mg I/mL) [14]. Comparing to previous work on this prototype dSi PCD CT by Crotty et al. (2022), an iodine density error of 4% was observed for 0.25 mg I/mL [17]. The same iodine concentration was not available in this work, but the closes was 0.2 mg I/mL, which demonstrated an iodine density percent error of −3.0%, which is better performance than was previously observed by Crotty et al. (2022) [17].

Overall, the prototype dSiPCD CT performs similar or better than some EID systems and the PCD CT system for some of the smaller iodine concentrations, and better than the commercially available EID and PCD CT systems for the larger iodine concentrations. Due to the prototype nature of this dSi PCD CT system, these results could change in the future.

This study has several limitations. First, the dSi PCD CT system used is a prototype and therefore the results may not accurately reflect a finalized commercially available version. Additionally, the corrections for scatter and beam hardening are not fully developed, and thus the accuracy and consistency of these measurements will likely improve. Second, the phantom used (MECT Gammex, Sun Nuclear, Middleton, WI) has a solid water background, which will introduce an iodine quantification error due to the difference between solid water and true water [10]. To account for this, a physics-based correction was applied to allow comparison with phantoms of true iodine and water. However, this correction is purely based on the physics of material decomposition and has not yet been tested on an iodine water phantom. Future work involves testing the accuracy of this correction method. Third, only two scanners were tested in this work and including more vendors and scanners could increase the applicability of this work. Finally, only one phantom size was used in this study, and it is well known that phantom size will affect iodine quantification accuracy [13–15]. Future work should include an examination of iodine density percent error over phantoms of several sizes and an investigation into how the performance on low density iodine inserts will translate into clinical practice.

## Conclusion

This work examined the accuracy of iodine quantification on a prototype dSi PCD CT over a range of iodine densities. Despite the limitations of the prototype, iodine accuracy was similar to or better than currently available EID and PCT CT systems. This demonstrates that the dSi PCD CT might allow for important clinical tasks involving iodine quantification. Improved iodine quantification in clinical practice could lead to improved characterization of incidental lesions, renal lesion identification, or other iodine quantification tasks such as perfusion.

## Data Availability

No datasets were generated or analysed during the current study.
